# Association between cognitive function in visuospatial‐construction and immediate memory domains with quality of life in patients with type 2 diabetes

**DOI:** 10.1002/alz70862_109857

**Published:** 2025-12-23

**Authors:** Ting Wen W Goh, Keven Ang, Serena Low, Su Chi Lim

**Affiliations:** ^1^ Raffles Institution, Singapore, Singapore Singapore; ^2^ Khoo Teck Puat Hospital (Alexandra Health Pte Ltd), Singapore, Singapore Singapore

## Abstract

**Background:**

Patients with type 2 diabetes (T2D) have increased susceptibility to cognitive impairment and experience poorer quality of life (QOL). It is unknown which domains of cognitive function are associated with QOL. We aimed to study the association between cognitive domains and QOL in patients with T2D.

**Method:**

This was a cross‐sectional study of 327 patients with T2D recruited from SMART2D cohort. Cognitive function was assessed using Repeatable Battery for Assessment of Neuropsychological Status (RBANS). The cognitive tool comprises five domains, namely immediate memory, delayed memory, visuospatial‐construction, language and attention. QOL was assessed using Euroqol EQ‐5D which covers visual analogue scale (VAS) and five domains in mobility, self‐care, usual activities, pain/discomfort and depression/anxiety. We examined the association between cognitive domains and QOL with linear and logistic regressions, adjusting for demographics, clinical parameters and medication.

**Result:**

The mean age was 61.5 ± 10.6 years. RBANS score in visuospatial‐construction domain was associated with higher EQ‐5D index score with adjusted coefficient 0.01 (95%CI 0.00 to 0.02; *p* = 0.037), and lower odds of problems in mobility, usual activities and self‐care domains with corresponding adjusted odds ratios (ORs) 0.96 (95%CI 0.93 to 0.98; *p* = 0.001), 0.95 (95%CI 0.92 to 0.99; *p* = 0.006) and 0.97 (95%CI 0.94 to 1.00; *p* = 0.041). RBANS score in immediate memory domain was associated with higher VAS with adjusted coefficient 1.47 (95%CI 0.19 to 2.74; *p* = 0.024).

**Conclusion:**

Higher cognitive function in visuospatial‐construction and immediate memory domains was associated with better QOL. The findings may pave the way for future interventions targeting specific cognitive function domains in an effort to improve QOL in patients with T2D.

